# Ventral Attention Network Resting State Functional Connectivity: Psychosocial Correlates among US Adolescents

**DOI:** 10.31586/jbls.2025.6208

**Published:** 2025-11-06

**Authors:** Shervin Assari, Alexandra Donovan, Babak Najand, Golnoush Akhlaghipour, Mario F Mendez

**Affiliations:** 1 Department of Psychiatry, Charles R Drew University of Medicine and Science, Los Angeles, CA, USA; 2 Department of Internal Medicine, Charles R Drew University of Medicine and Science, Los Angeles, CA, USA; 3 Department of Public Health, Charles R Drew University of Medicine and Science, Los Angeles, CA, USA; 4 Marginalization-related Diminished Returns Center, Los Angeles, CA, USA; 5 Montefiore Einstein’s Adult Neurology Residency Program, Bronx, New York, USA; 6 Department of Neurology, University of California Los Angeles (UCLA), Los Angeles, CA, USA; 7 Department of Psychiatry & Biobehavioral Sciences, University of California Los Angeles (UCLA), Los Angeles, CA, USA

**Keywords:** Ventral Attention Network, Resting-State fMRI, Adolescence, Socioeconomic Status, Discrimination, ABCD Study

## Abstract

**Background:**

Resting-state functional MRI (rsfMRI) provides insights into large-scale brain network organization associated with cognitive control, emotion regulation, and attentional processes. The ventral attention network (VAN) is a key salience-driven network that supports attentional re-orienting to behaviorally relevant stimuli. However, little is known about how VAN resting state functional connectivity varies by demographic, socioeconomic, psychosocial, and behavioral factors during early adolescence.

**Objective:**

To examine associations between VAN rsfMRI connectivity and multiple demographic, socioeconomic, psychosocial, and behavioral characteristics.

**Methods:**

Data came from the baseline and early follow-up waves of the Adolescent Brain Cognitive Development (ABCD) Study. The analytic sample included youth with high-quality baseline rsfMRI data and complete socioeconomic and psychosocial measures. The primary outcome was mean resting-state functional connectivity within the VAN across subcortical and cortical regions of interest (ROIs). Bivariate correlations were computed between VAN connectivity and demographic (age, sex, puberty, race/ethnicity), socioeconomic (income, parental education, marital status, neighborhood income), psychosocial (trauma, discrimination, financial difficulty), trait (impulsivity), and behavioral variables (body mass index, depression, suicide, prodromal symptoms, and substance use). Unadjusted bivariate correlations and adjusted logistic regressions were used for data analysis.

**Results:**

VAN connectivity showed small but significant correlations with multiple contextual factors. Higher household income, parental education, and neighborhood affluence were associated with greater connectivity, whereas Black race and Hispanic ethnicity were related to lower connectivity. Youth reporting higher discrimination and financial difficulty exhibited weaker VAN connectivity. Greater VAN connectivity was negatively associated with impulsive reward-driven trait (drive), prodromal symptoms, BMI, and marijuana and alcohol use. Associations between VAN connectivity and suicide, depression, marijuana use, and alcohol use remained significant in age and sex adjusted models.

**Conclusions:**

VAN connectivity reflects subtle neural correlates of socioeconomic and psychosocial context in early adolescence. Our results underscore the importance of integrating structural and contextual factors in interpreting brain-behavior associations across diverse populations. These findings are suggestive of stable socioeconomic and psychosocial correlates of network efficiency.

## 1. Introduction

Adolescence represents a critical developmental period characterized by extensive neural maturation and increasing sensitivity to environmental and social contexts [1-3]. During this stage, large-scale brain networks that support attention, executive function, and emotion regulation undergo rapid reorganization. Among these, the ventral attention network (VAN)—also referred to as the salience-driven attentional control system—plays a central role in detecting and orienting toward behaviorally relevant stimuli, switching between externally and internally directed attention, and supporting flexible adaptation to environmental demands [4-7]. Functional connectivity within this network, particularly in resting-state fMRI (rsfMRI), has been associated with attentional performance, impulse regulation, and susceptibility to stress-related psychopathology [4,5,7-12].

Emerging research has established that social and economic resources during childhood and adolescence are associated with measurable differences in brain structure and function [13]. Higher socioeconomic status (SES)—often indexed by income, parental education, or neighborhood affluence—has been linked with greater cortical thickness, larger hippocampal and amygdala volumes, and stronger connectivity in networks supporting cognitive control and learning [14-20]. These associations are thought to reflect differences in exposure to cognitive stimulation, nutrition, and stress across developmental contexts. Because neural systems are shaped through ongoing interactions with social environments, understanding how VAN connectivity relates to structural and contextual factors such as SES, discrimination, and psychosocial adversity may help elucidate the mechanisms linking social inequality with neurodevelopmental outcomes.

The Adolescent Brain Cognitive Development (ABCD) Study provides a unique opportunity to investigate these dynamics on a population scale [21-33]. This large, longitudinal study includes racially and socioeconomically diverse U.S. adolescents with harmonized multimodal MRI and comprehensive assessments of family, neighborhood, and psychosocial environments. While prior ABCD findings have linked SES and discrimination to neural structure, connectivity, and cognition, the ventral attention network remains underexamined in this context [34,35]. VAN’s role as a salience-processing system makes it particularly sensitive to stress and social context. Alterations in VAN connectivity have been reported among youth exposed to adversity, trauma, and poverty, yet few studies have explored how everyday socioeconomic and psychosocial experiences—including discrimination, financial difficulty, and family context—relate to its intrinsic functional organization.

In addition to social factors, VAN connectivity may also show behavioral and health-related correlates. Lower VAN coherence has been associated with difficulties in sustained attention, greater impulsivity, and risk for substance use and mood disorders [36-38]. Conversely, stronger VAN integration may support self-regulatory processes that protect against maladaptive behaviors. Because adolescence is a period marked by rising rates of obesity, depression, and early substance experimentation, identifying neurobiological correlates of these outcomes may inform early prevention strategies. Understanding whether VAN connectivity varies systematically by SES, race, and psychosocial adversity may also illuminate potential neural mechanisms underlying population-level disparities in health and behavior.

Given these gaps, the current study examined correlates of resting-state VAN connectivity using data from the ABCD Study. We focused on cross-sectional associations at baseline between VAN rsfMRI connectivity and a wide range of demographic, socioeconomic, psychosocial, and behavioral variables, and explored longitudinal patterns for discrimination, body mass index (BMI), and substance use. We hypothesized that higher SES would be associated with stronger VAN connectivity overall. We also anticipated that VAN connectivity would be associated with BMI, depressive symptoms, and substance use. This work aims to extend current understanding of how social and economic contexts shape adolescent neural functioning.

## 2. Methods

### Study Design and Participants

This study utilized data from the Adolescent Brain Cognitive Development (ABCD) Study, a large, ongoing, multi-site, longitudinal cohort designed to examine environmental, social, and biological influences on adolescent brain and behavioral development. Baseline data were collected from more than 11,800 children aged 9–10 years across 21 research sites in the United States. Participants were recruited through probability sampling of schools to ensure sociodemographic diversity reflective of the national population.

The current analysis was restricted to participants with high-quality resting-state functional MRI (rsfMRI) data and complete information on key socioeconomic and psychosocial variables at baseline. Exclusion criteria included missing or poor-quality imaging data, scanner artifacts, or incomplete covariate information. The final analytic sample consisted of 7,448 youth. Longitudinal analyses used follow-up data from subsequent waves for measures of discrimination, body mass index (BMI), and substance use, allowing examination of the persistence of these correlates over time.

### Measures

#### Resting-State fMRI: Ventral Attention Network Connectivity:

Resting-state fMRI data were processed using the ABCD Study’s standardized pipeline, which includes correction for head motion, slice timing, and spatial normalization. Functional connectivity estimates were derived from the ventral attention network (VAN), a system implicated in salience detection and reorienting of attention. For each participant, mean Fisher z-transformed correlation coefficients were calculated between the VAN and multiple ASEG-defined subcortical regions of interest (ROIs). These included the hippocampus, amygdala, caudate, putamen, pallidum, thalamus, cerebellar cortex, ventral diencephalon, and brainstem.

#### Demographic and Developmental Factors:

Participants’ age (in years and months), sex (male/female), race/ethnicity, immigration status, and pubertal status were included as demographic and biological covariates. Pubertal development was categorized as pre-pubertal or early to mid-puberty using the Pubertal Development Scale, which captures self-reported physical changes consistent with early adolescence. Personality factors were assessed via the BIS/BAS scale, reporting on drive, reward responsiveness, and the UPPS-P scale reporting on positive urgency.

#### Socioeconomic Indicators:

Socioeconomic position was operationalized using multiple indicators collected at baseline. Household income was reported in categorical ranges, parental education was coded as the highest completed level by either parent, and marital/partnered household status was defined as living with married or cohabitating caregivers. Neighborhood income was estimated using census tract–based median household income linked to participants’ residential addresses.

#### Psychosocial Adversity and Family Context:

Psychosocial adversity measures included financial difficulty, trauma exposure, and perceived discrimination. Financial difficulty reflected parental reports of trouble meeting basic needs, while trauma exposure was based on a standardized count of adverse life events. Discrimination was assessed using youth self-report of unfair treatment, with follow-up data available longitudinally. Family emotional context was characterized by two youth-reported subscales: family love and support and family conflict. Higher scores indicated stronger perceived affection and higher conflict, respectively.

### Behavioral and Clinical Outcomes

Behavioral and health-related outcomes included BMI, depression, prodromal symptom severity, suicide risk, and substance use behaviors. BMI was calculated from measured height and weight. Depression was derived from KSADS. Prodromal symptoms and psychosocial impairment were indexed from structured assessments capturing early markers of risk for psychopathology. Mania was assessed using the 7-UP inventory. Substance use variables included any reported nicotine, marijuana, or alcohol use at baseline or follow-up.

### Statistical Analysis

Analyses were conducted using SPSS version 29. All variables were inspected for normality and missingness. The primary analytic approach involved bivariate Pearson correlations between average VAN connectivity and each demographic, socioeconomic, psychosocial, and behavioral variable. Correlations were computed using two-tailed tests, with significance thresholds set at p < 0.05 and p < 0.01 to denote nominal and robust associations, respectively.

To evaluate the internal consistency of the VAN connectivity composite, a reliability analysis was performed across the 21 subcortical connectivity indicators, yielding corrected item-total correlations and Cronbach’s alpha coefficients. The analysis indicated moderate internal consistency across 21 VAN–ASEG connectivity indicators (Cronbach’s α = 0.535), suggesting partial but meaningful coherence within this network-level composite. Subsequent analyses used the average VAN rsfMRI connectivity score as the primary outcome variable.

For longitudinal analyses, follow-up measures of discrimination, BMI, and substance use were descriptively examined in relation to baseline VAN connectivity. The goal was to assess whether early neural patterns remained meaningfully related to later psychosocial and behavioral outcomes. No causal or directional inference was made; rather, findings were interpreted as indicative of potential social and behavioral correlates of VAN functional organization.

After unadjusted bivariate correlations, we used logistic regression to control for the effects of age and sex, testing for associations between VAN connectivity with marijuana use, alcohol use, depression, and suicide.

### Ethical Considerations

All data collection in the ABCD Study was approved by the Institutional Review Boards of participating institutions. Written informed consent was obtained from parents or guardians, and assent was obtained from participating youth. The present secondary data analysis was approved under data use agreement guidelines and complied with all ethical standards for human subjects research.

## 3. Results

### Sample Characteristics

The analytic sample included 7,879 participants with available data at baseline. Most youth were between 9 and 10 years of age, with approximately half (49.5%) aged 9 and 50.2% aged 10 years. A small proportion (0.1%) were aged 8 years or slightly above 9 years, reflecting the narrow age range targeted by the ABCD baseline recruitment. In terms of sex distribution, the sample was nearly evenly split, with 49.6% girls and 50.4% boys. Regarding pubertal status, among the 6,300 participants with available data, 27.6% were prepubertal and 72.4% had entered puberty, indicating that most participants were in early to mid-pubertal stages. Racial and ethnic composition was predominantly non-Black and non-Hispanic. Based on the available data, 79.8% identified as White and 20.2% as Black, while 80.6% were non-Hispanic and 19.4% identified as Hispanic or Latino. Only a small portion of participants were immigrants (3.0%), suggesting that most were U.S.-born.

Family structure and socioeconomic indicators reflected considerable variation. About 76.2% of youth lived in married or partnered households, while 23.8% lived in single or non-partnered homes. Financial strain was relatively uncommon but notable: 19.1% of families reported some level of financial difficulty, and 80.9% did not. Exposure to traumatic experiences was reported by approximately 34.9% of youth, while 65.1% had no trauma history based on DSM-aligned indicators. Clinical indicators showed that past psychiatric symptoms were relatively rare at this age. Among those with data, 2.3% reported a past suicide attempt, and 1.7% met criteria for a past major depressive disorder (MDD). Missingness for these variables was substantial, reflecting the young age of the sample and the low base rate of clinical events. Regarding substance use, 3.9% reported any nicotine use, 2.0% reported marijuana use, and 34.2% reported alcohol use. Thus, while nicotine and marijuana use were relatively uncommon, alcohol exposure appeared substantially higher, consistent with patterns of early experimentation observed in the ABCD cohort.

Table 1 summarizes the continuous study variables. The mean ventral attention network rsfMRI connectivity was 0.035 (SD = 0.050), indicating small but measurable variability in average network correlation strength across participants. The mean age in months of the sample was approximately 119 months (SD = 7.49), corresponding to just under 10 years, consistent with the target age range of the ABCD baseline cohort.

Socioeconomic indicators showed broad variation. The mean parental education was 16.82 years (SD = 2.64), suggesting that on average caregivers had completed some college or postsecondary education. The median neighborhood family income was $79,006 (SD = $35,795), reflecting substantial geographic and socioeconomic diversity.

Psychosocial measures reflected generally supportive but heterogeneous family environments. The average Family Environment Scale (FES) Love score was 3.23 (SD = 1.30), whereas FES Conflict averaged 0.71 (SD = 1.00), suggesting that most participants perceived moderate family warmth and low conflict overall. Perceived discrimination at Year 1 was low on average (M = 1.18, SD = 0.41), though the observed variability indicates that some youth already reported experiences of unfair treatment during early adolescence.

Behavioral and psychological measures demonstrated expected early-adolescent ranges. The 7UP sum score, indexing positive mood and activity, averaged 1.97 (SD = 2.62). BIS/BAS Drive (M = 4.00, SD = 2.97) and Reward Responsiveness (M = 10.92, SD = 2.90) reflected moderate motivational levels. UPPS-P Positive Urgency had a mean of 7.94 (SD = 2.94), indicating individual differences in impulsive responses to positive affect.

Clinical and health indicators were largely within normal limits. The mean Prodromal Psychosis Scale score was 2.02 (SD = 2.55), with a mean severity score of 5.38 (SD = 9.83), suggesting generally low subclinical symptom burden. The average body mass index (BMI) at Year 1 was 19.23 (SD = 4.31), consistent with age-appropriate healthy weight ranges for this developmental stage.

### Item-Level Statistics

As shown by Table 2, item-total correlations ranged from −0.028 to 0.368, indicating variability in how strongly each regional correlation contributed to the overall scale. The VAN–left hippocampus (r = 0.368) showed the strongest relationship with the overall composite, followed by VAN–brainstem (r = 0.321) and VAN–left caudate (r = 0.325). These three regions appear to align most consistently with the broader ventral attention network. In contrast, several VAN correlations demonstrated weaker or even negligible item-total associations, particularly those involving the left pallidum (r = −0.003), brainstem (r = −0.028), and left thalamus-proper (r = 0.039).

### Alpha if Item Deleted

Cronbach’s alpha if item deleted ranged from 0.482 to 0.554, indicating that removal of any single item would not meaningfully improve internal consistency. The scale’s reliability remained relatively stable, suggesting that no single region disproportionately affected the composite measure’s internal coherence. The 21-item scale demonstrated moderate reliability (α = .535).

### Correlates of Ventral Attention Network rsfMRI Connectivity

As shown in Table 3, the average VAN functional connectivity (rsfMRI) showed several small but statistically significant correlations with demographic, socioeconomic, psychosocial, and behavioral variables.

### Demographic factors

VAN rsfMRI connectivity was slightly lower among participants of older chronological age (r = −0.026, p < 0.05 for age in years; r = −0.029, p < 0.05 for age in months) and among those at more advanced pubertal stages (r = −0.031, p < 0.05). No meaningful associations were observed with sex (r = 0.012, ns).

### Socioeconomic indicators

Higher household income (r = 0.101, p < 0.01), higher parental education (r = 0.104, p < 0.01), being in a married or partnered household (r = 0.099, p < 0.01), and residence in higher-income neighborhoods (r = 0.097, p < 0.01) were all associated with greater VAN rsfMRI connectivity. Conversely, Black race (r = −0.145, p < 0.01) and Hispanic ethnicity (r = −0.059, p < 0.01) were associated with lower connectivity in this bivariate comparison.

### Psychosocial adversity

Greater financial difficulty (r = −0.071, p < 0.01) and discrimination (r = −.044, p < .01) were inversely correlated with VAN connectivity. A marginal negative association was also found for trauma exposure (r = −0.023, p < 0.05).

### Family and emotional context

rsfMRI was not significantly related to perceived love (r = −0.003, ns) or conflict (r = −0.001, ns) within the household.

### Motivational and cognitive-affective traits

Small negative correlations emerged with measures of positive drive (r = −0.041, p < 0.01), reward responsiveness (r = −0.031, p < 0.05), and positive urgency (r = −0.045, p < 0.01), suggesting that higher VAN connectivity may relate to somewhat lower impulsive reward-driven tendencies.

### Clinical and behavioral outcomes

Weaker connectivity was also associated with greater prodromal psychosis symptoms (r = −0.035, p < 0.05) and greater prodromal symptom severity (r = −0.052, p < 0.01). Lower rsfMRI values were modestly related to higher body mass index (r = −0.088, p < 0.01) and higher depressive symptoms (r = −0.050, p < 0.01). Small inverse associations were observed for suicide risk (r = −0.025, p < 0.05), and for substance use, particularly nicotine (r = −0.032, p < 0.01), though correlations with marijuana (r = 0.023, p < 0.05) and alcohol (r = −0.014, ns) were minimal.

### Associations Between Ventral Attention Network Connectivity and Clinical and Behavioral Outcomes

Binary logistic regressions were conducted to examine whether ventral attention network (VAN) rsfMRI connectivity predicted the likelihood of suicide attempt, past major depressive disorder (MDD), marijuana use, and alcohol use, after adjusting for age and sex.

### Suicide Attempt

As shown in Table 5, higher VAN rsfMRI connectivity was significantly associated with a lower likelihood of suicide attempt (B = −6.22, SE = 2.15, Wald = 8.34, p = 0.004). The odds ratio (Exp(B) = 0.002, 95% CI [0.000, 0.136]) indicated that each unit increase in VAN connectivity was linked with a markedly reduced probability of having ever attempted suicide. Neither age (p = 0.407) nor sex (p = 0.416) significantly predicted suicide attempt in this model.

### Major Depressive Disorder (MDD)

Similarly, higher VAN connectivity was associated with lower odds of past MDD diagnosis (B = −3.75, SE = 1.85, Wald = 4.11, p = 0.043). The corresponding odds ratio (Exp(B) = 0.024, 95% CI [0.001, 0.881]) suggested that stronger VAN connectivity was related to a substantially lower likelihood of having a history of MDD. Age and sex were not significant covariates in this model (p > 0.29 for both) (Table 5).

### Marijuana Use

VAN connectivity also demonstrated a robust inverse association with marijuana use (B = −4.57, SE = 1.57, Wald = 8.48, p = 0.004). The odds ratio (Exp(B) = 0.010, 95% CI [0.000, 0.224]) indicated that higher VAN connectivity corresponded with sharply lower odds of having used marijuana. In contrast, both age (B = 0.062, p < 0.001) and male sex (B = 0.367, p = 0.027) were significant positive predictors, showing that older and male adolescents were more likely to report marijuana use (Table 5).

### Alcohol Use

In contrast to the other outcomes, VAN connectivity showed a positive association with alcohol use (B = 1.04, SE = 0.48, Wald = 4.70, p = 0.030). The odds ratio (Exp(B) = 2.82, 95% CI [1.11, 7.19]) indicated that higher connectivity predicted increased odds of alcohol consumption. Age (B = 0.021, p < 0.001) and male sex (B = 0.176, p < 0.001) were also significant predictors, with older and male participants more likely to report alcohol use (Table 5).

## 4. Discussion

This study examined the socioeconomic, psychosocial, and behavioral correlates of ventral attention network (VAN) resting-state functional connectivity among early adolescents in the ABCD Study, with longitudinal exploration of discrimination, BMI, and substance use. Several modest but statistically significant associations were identified, suggesting that VAN connectivity reflects a neural characteristic that varies systematically across social and behavioral dimensions. Although the magnitudes of these effects were small, the consistency of associations across diverse domains underscores the sensitivity of this network to contextual and developmental influences.

Higher household income, parental education, and neighborhood income were all associated with stronger VAN connectivity. These findings are consistent with prior research linking socioeconomic resources to enhanced neural integration in networks supporting cognitive control, attention, and emotion regulation. Youth from more resource-rich environments are often exposed to higher cognitive stimulation, safer and more organized surroundings, better nutrition, and reduced exposure to chronic stress—all of which can support healthier neurodevelopmental trajectories. Stronger VAN connectivity may therefore reflect more efficient coordination of attentional and salience processing systems among adolescents with greater social advantage.

Conversely, lower connectivity among youth from less advantaged contexts may indicate the neural consequences of sustained exposure to stress, instability, or reduced enrichment opportunities. Chronic socioeconomic stress has been linked to alterations in brain regions that overlap with the VAN, including the anterior insula, cingulate cortex, and subcortical structures, all of which play roles in salience detection and attentional reorientation [39-41]. These findings highlight that neural systems central to attentional control may be particularly sensitive to environmental and socioeconomic variability during adolescence.

Indicators of psychosocial adversity—including discrimination, financial difficulty, and trauma exposure—were each related to lower VAN connectivity. The VAN is functionally responsible for identifying and prioritizing salient stimuli, including potential threats, and coordinating the switch between internally and externally directed attention. These findings are consistent with prior evidence linking social adversity to dysregulation of attention network [42-44]. Repeated activation of this network under stressful or threatening conditions, such as discrimination or social exclusion, may lead to reduced neural efficiency or altered baseline connectivity. For example, exposure to discrimination and chronic stress during sensitive developmental periods may shape neural organization through repeated engagement of vigilance-related circuits, promoting a state of hyper-alertness that could reduce network coherence at rest.

Lower VAN connectivity was associated with higher body mass index (BMI), greater depressive and prodromal symptoms, higher drive and reward responsivity, increased positive urgency and mania symptoms, and elevated risk for substance use, particularly marijuana. These associations, though small, are consistent with the VAN’s role in integrating interoceptive and affective signals with cognitive control. Reduced VAN connectivity may reflect diminished efficiency in coordinating attention toward adaptive cues or regulating responses to stress and reward, processes that are relevant to both emotional regulation and behavioral self-control. The negative associations between VAN connectivity and depressive symptoms align with prior imaging studies showing decreased salience and attention network coherence in adolescents with mood symptoms [45-47]. Similarly, links to marijuana and other substances may reflect early differences in neural systems involved in reward anticipation and impulse control. It remains unclear whether lower connectivity represents a predisposing vulnerability for these behaviors or a neurobiological consequence of them, but the findings underscore the VAN’s potential importance in the regulation of attention, emotion, and reward.

Taken together, these findings indicate that VAN connectivity is modestly shaped by a combination of socioeconomic advantage, psychosocial strain, and behavioral regulation. The VAN’s sensitivity to environmental variation may reflect its position at the intersection of cognitive and affective processing. During adolescence, when attentional control and emotional reactivity are still maturing, environmental exposures—whether enriching or stressful—can influence the efficiency of communication between VAN regions and subcortical structures. The present results suggest that adolescents experiencing higher social and economic stability exhibit more cohesive VAN connectivity, whereas those exposed to adversity may show attenuated network organization, potentially reflecting adaptive responses to stress or reduced capacity for attentional modulation.

Across logistic regression models, VAN rsfMRI connectivity displayed distinct patterns depending on outcome type. Higher connectivity was inversely associated with self-reported suicide attempt, past MDD, and marijuana use—suggesting that stronger VAN coherence may be linked with lower risk for self-harm, mood disturbance, and substance initiation. However, the positive association with alcohol use suggests that not all behavioral outcomes follow the same pattern, possibly reflecting different social or developmental contexts surrounding early alcohol exposure. A previous analysis of ABCD Study data identified an increase in risk of alcohol use among adolescents with higher family income and parental education level, thus the positive correlation of VAN connectivity with alcohol may be related to the positive correlation of connectivity with SES. [42,48] Age and sex effects were consistent, with older and male adolescents showing higher likelihood of substance use overall. These regression findings collectively indicate that VAN connectivity is modestly but significantly related to a range of emotional and behavioral outcomes in early adolescence, emphasizing its potential role as a neural correlate of risk and resilience across multiple domains.

These results contribute to growing evidence that social context is a determinant of neural organization [49,50]. The associations between socioeconomic disadvantage, discrimination, and VAN connectivity emphasize the need to integrate environmental and structural variables into neurodevelopmental research. By situating brain function within a broader ecological framework, this work highlights that neural measures are not purely biological traits but are influenced by social and material conditions [51].

From a public health perspective, interventions aimed at improving family stability, reducing discrimination, and promoting positive school and neighborhood environments could support both psychosocial outcomes and healthy neurodevelopment. Policies that mitigate chronic stress exposure in youth—such as those enhancing economic security or reducing exposure to neighborhood violence—may help maintain or restore optimal connectivity within networks supporting attention and regulation.

### Limitations

Several limitations should be noted. The analyses were largely cross-sectional, limiting causal inference. Although longitudinal data on discrimination, BMI, and substance use were considered, further follow-up is required to clarify developmental directionality. The experiences of discrimination reported could not have directly impacted VAN connectivity, as the connectivity was measured one year prior to the reported discrimination. The internal consistency of the VAN composite was moderate (Cronbach’s α = .535), suggesting some heterogeneity among the regional connectivity indicators. Despite the large sample size, the effect sizes were small, indicating that individual differences in VAN connectivity explain a limited proportion of variance in psychosocial and behavioral outcomes. Moreover, while the ABCD Study’s national sampling improves representativeness, measures of discrimination, trauma, and family dynamics rely on self-report and may not fully capture contextual complexity.

### Future Directions

Future work should explore multivariate and longitudinal models to determine how VAN connectivity evolves with changing socioeconomic and psychosocial experiences, as many factors explored in the current study are also significantly associated with SES. Mediation and moderation analyses could clarify whether discrimination or financial strain influences behavioral outcomes through alterations in attentional network efficiency. Integrating structural MRI, task-based fMRI, and physiological stress markers may offer a more complete understanding of how environment and experience shape network-level function. Additionally, replication across culturally diverse and international samples could determine whether these associations reflect universal developmental processes or context-specific dynamics.

This study examined the socioeconomic, psychosocial, and behavioral correlates of ventral attention network (VAN) resting-state functional connectivity among early adolescents in the ABCD Study, with longitudinal exploration of discrimination, BMI, and substance use. Several modest but statistically significant associations were identified, suggesting that VAN connectivity reflects a neural characteristic that varies systematically across social and behavioral dimensions. Although the magnitudes of these effects were small, the consistency of associations across diverse domains underscores the sensitivity of this network to contextual and developmental influences.

## 5. Conclusions

In summary, this study demonstrates that ventral attention network connectivity during early adolescence is modestly but consistently associated with socioeconomic conditions, psychosocial adversity, and behavioral outcomes. Greater social and economic stability corresponded with stronger network integration, whereas adversity and stress-related exposures were linked with weaker connectivity. These results suggest that VAN organization is sensitive to the broader environments in which youth develop and underscore the importance of addressing structural and contextual factors to support healthy brain development.

### Ethics Statement

This study used publicly available, deidentified data from the Adolescent Brain Cognitive Development (ABCD) Study, a multisite, longitudinal study approved by the Institutional Review Boards (IRBs) at all 21 participating institutions. All ABCD participants provided written assent, and their parents or legal guardians provided written informed consent prior to participation. The present secondary data analysis involved only deidentified data and therefore did not constitute human subjects research as defined by federal regulations. Accordingly, the current analysis was deemed IRB exempt. All procedures were conducted in accordance with the Declaration of Helsinki and adhered to relevant ethical guidelines and regulations governing research with human participants.

## Figures and Tables

**Table 1.** Descriptive Statistics

	Mean	Std. Deviation
rsfMRI	0.0346	0.05009
Age (months)	119.3452	7.48782
Parent education years highest	16.8174	2.63739
Residential history Median family income	79006.72	35795.194
Discrimination Yr1	1.1783	0.40610
FES Family Love	3.2309	1.29815
FES Family Conflict	0.7092	.99748
7UP sum	1.97	2.620
BIS/BAS Drive	4.00	2.973
BIS/BAS Reward Responsiveness	10.92	2.897
UPPS-P Positive Urgency	7.94	2.936
Prodromal Psychosis Scale	2.02	2.553
Prodromal Psychosis: Severity Score	5.38	9.828
BMI Yr 1	19.2323	4.31275

FES: Family Environment Scale; BMI: Body Mass Index; BIS BAS: Behavioral Inhibition System / Behavioral Activation System; UPPS-P: UPPS-P Impulsive Behavior Scale for Children; rsfMRI: average functional connectivity of the ventral attention network

**Table 2.** Item-Total Statistics

	Scale Mean if Item Deleted	Scale Variance if Item Deleted	Corrected Item-Total Correlation	Cronbach’s Alpha if Item Deleted
VAN connectivity with ASEG ROI left-hippocampus	.631160	.931	.368	.482
VAN connectivity with ASEG ROI brain-stem	.642460	1.043	.321	.512
VAN connectivity with ASEG ROI left-caudate	.703003	1.003	.325	.501
VAN connectivity with ASEG ROI right-accumbens-area	.720659	1.013	.264	.509
VAN connectivity with ASEG ROI right-amygdala	.612354	.986	.158	.527
VAN connectivity with ASEG ROI right-hippocampus	.699230	1.017	.098	.540
VAN connectivity with ASEG ROI right-pallidum	.755613	1.057	.091	.534
VAN connectivity with ASEG ROI right-putamen	.636338	1.033	.093	.538
VAN connectivity with ASEG ROI right-caudate	.683847	1.064	.211	.522
VAN connectivity with ASEG ROI right-thalamus-proper	.761521	1.089	.005	.544
VAN connectivity with ASEG ROI left-ventral dc	.703157	1.036	.180	.521
VAN connectivity with ASEG ROI left-amygdala	.679038	1.036	.169	.523
VAN connectivity with ASEG ROI left-hippocampus	.670506	.982	.307	.499
VAN connectivity with ASEG ROI brain-stem	.792453	1.090	−.028	.554
VAN connectivity with ASEG ROI left-pallidum	.706664	1.089	−.003	.546
VAN connectivity with ASEG ROI left-caudate	.765909	1.021	.098	.539
VAN connectivity with ASEG ROI left-thalamus-proper	.737661	1.074	.039	.541
VAN connectivity with ASEG ROI left-cerebellum-cortex	.652511	1.039	.081	.540

ASEG: Automatic Subcortical Segmentation; VAN: Ventral Attention Network; ROI: Region of Interest

**Table 3.** Bivariate Correlations of variables with VAN connectivity

Bivariate Correlation (Pearson)	rsfMRI VAN
Age (years)	−0.026*
Age (months)	−0.029*
Sex (male)	0.012
Puberty	−0.031*
Income	0.101**
Parental Education	0.104**
Married/Partnered Household	0.099**
Neighborhood Income	0.097**
Race (Black)	−0.145**
Ethnicity (Hispanic)	−0.059**
Immigration status (Immigrant)	0.007
Trauma (any)	−0.023*
Financial Difficulty	−0.071**
Discrimination	−0.044**
Family Love	−0.003
Family Conflict	−0.001
7-UP (mania)	−0.050*
BIS/BAS (Drive)	−0.041**
BIS/BAS (Reward Responsiveness)	−0.031*
UPPS-P (Positive Urgency)	−0.045**
Prodromal Psychosis Symptoms	−0.035*
Prodromal Symptom Severity	−0.052**
BMI	−0.088**
Suicide	−0.050**
Major Depression Diagnosis	−0.025*
Nicotine Use	−0.014
Marijuana Use	−0.032**
Alcohol Use	0.023*

*. Correlation is significant at the 0.05 level (2-tailed).

**. Correlation is significant at the 0.01 level (2-tailed). rsfMRI= ventral attention network (VAN) resting-state functional connectivity.

**Table 4.** Bivariate correlations across study variables

	rsfMRI	Age 10	Age Month	Male	Puberty	Income	Parental Education	Married/Partnered HH	Neighborhood income	Black	Hispanic Ethnicity	Immigrant	Trauma	Financial Difficulty	Discrimination	Love	Conflict	7Up	Drive	Reward	+ Urgency	Prodromal Psychosis Symptoms	Prodromal Symptoms Severity	BMI	Suicide	MDD	Nicotine	Marijuana	Alcohol
rsfMRI	1	−.026*	−.029*	0.012	−.031*	.101**	.104**	.099**	.097**	−.145**	−.059**	0.007	−.023*	−.071**	−.044**	−0.003	−0.001	−.050*	−.041**	−.031*	−.045**	−.035*	−.052**	−.088**	−.050**	−.025*	−0.014	−.032**	.023*
Age 10		1	.855**	.025*	.116**	.031**	0.012	0.004	.040**	−0.007	−.023*	−0.006	0.009	−0.021	−.036**	0.010	0.003	−.077**	0.022	0.004	−.026*	−.045**	−.047**	.100**	0.003	0.021	.066**	.056**	.070**
Age Month			1	.024*	.133**	.028*	0.011	−0.004	.049**	−0.009	−.030**	−0.005	0.012	−0.008	−.045**	0.010	0.012	−.075**	0.012	−0.003	−.043**	−.060**	−.060**	.103**	−0.015	0.014	.075**	.062**	.076**
Male				1	−.092**	0.003	0.011	0.009	0.011	−.031**	−0.012	0.000	−0.018	0.009	.067**	−.045**	.048**	.076**	.099**	.054**	.079**	−0.008	0.007	−0.029	0.014	0.012	0.006	.026*	.043**
Puberty					1	−.060**	−.056**	−.047**	−.072**	.098**	0.023	.031*	.052**	.027*	.073**	0.021	−0.003	.086**	0.015	.041**	.054**	.076**	.107**	.144**	.041*	.030*	.035**	.025*	.029*
Income						1	.605**	.507**	.526**	−.416**	−.239**	−.044**	−.110**	−.459**	−.201**	.033**	−.100**	−.168**	−.161**	−.111**	−.142**	−.089**	−.148**	−.204**	−.111**	−.043**	−.078**	−.067**	.143**
Parental Education							1	.257**	.447**	−.271**	−.314**	.034**	−.063**	−.303**	−.147**	−0.010	−.055**	−.152**	−.141**	−.081**	−.114**	−.102**	−.151**	−.190**	−.047**	−.026*	−.058**	−.052**	.150**
Married/Partnered HH								1	.247**	−.376**	−.056**	0.017	−.125**	−.251**	−.103**	.023*	−.075**	−.099**	−.094**	−.077**	−.112**	−.057**	−.102**	−.133**	−.081**	−.033**	−.049**	−.036**	.038**
Neighborhood income									1	−.337**	−.228**	−0.001	−.092**	−.273**	−.152**	−0.012	−.051**	−.148**	−.142**	−.104**	−.110**	−.090**	−.126**	−.180**	−.067**	−.030*	−.040**	−.054**	.145**
Black										1	−.086**	−0.002	.088**	.270**	.189**	−.033**	.082**	.122**	.148**	.128**	.130**	.074**	.146**	.154**	.070**	.053**	0.009	0.019	−.122**
Hispanic Ethnicity											1	.095**	−0.003	.088**	.063**	.057**	−.035**	.106**	.085**	.055**	0.013	.067**	.088**	.161**	0.028	0.006	0.020	.026*	−.045**
Immigrant												1	−0.013	−0.016	0.006	.023*	−.032**	0.015	.029*	0.005	−0.007	−0.021	−0.021	0.016	0.011	−0.008	−0.012	−0.003	−0.006
Trauma													1	.151**	.061**	−.059**	.112**	.069**	0.021	0.020	.043**	.059**	.066**	.047**	.047*	.040**	.071**	.031**	0.007
Financial Difficulty														1	.139**	−.105**	.163**	.114**	.084**	.058**	.104**	.083**	.110**	.129**	.082**	.055**	.057**	.036**	−.057**
Discrimination															1	−.023*	.061**	.311**	.122**	.036**	.147**	.212**	.243**	.098**	.109**	.087**	.061**	.046**	−.028*
Love																1	−.465**	−0.025	−.043**	−0.019	−.050**	−0.014	−0.016	−0.004	−0.016	−0.023	−0.020	−0.020	−.026*
Conflict																	1	0.018	.060**	.032*	.052**	0.025	.035**	0.008	.068**	0.009	.037**	.030**	−0.013
7Up																		1	.^c^	.^c^	.^c^	.475**	.521**	.048*	.228**	.129**	.088**	0.024	0.040
Drive																			1	.451**	.234**	.115**	.146**	.100**	.046*	.066**	.032*	0.011	−0.012
Reward																				1	.151**	.113**	.138**	.077**	0.026	.059**	0.017	0.009	−0.005
+ Urgency																					1	.171**	.220**	.102**	.077**	.083**	.049**	.044**	0.024
Prodromal Psychosis Symptoms																						1	.965**	0.029	.114**	.194**	.058**	.041**	.043**
Prodromal Symptoms Severity																							1	.069**	.157**	.217**	.072**	.045**	.063**
BMI																								1	.057*	0.042	.107**	.041*	−0.016
Suicide																									1	.043*	.087**	.048**	0.026
MDD																										1	.033**	0.008	0.011
Nicotine																											1	.291**	.145**
Marijuana																												1	.101**
Alcohol																													1

*. Correlation is significant at the 0.05 level (2-tailed).

**. Correlation is significant at the 0.01 level (2-tailed). rsfMRI= ventral attention network (VAN) resting-state functional connectivity

**Table 5.** Logistic regressions

	B	S.E.	Wald	df	Sig.	Exp(B)	95% CI	for Exp
Suicide								
rsfMRI	−6.219	2.154	8.337	1	0.004	0.002	0.000	0.136
Age months	−0.013	0.016	0.687	1	0.407	0.987	0.956	1.018
Male	0.197	0.242	0.662	1	0.416	1.217	0.758	1.955
Constant	−2.078	1.933	1.156	1	0.282	0.125		
MDD								
rsfMRI	−3.748	1.848	4.114	1	0.043	0.024	0.001	0.881
Age months	0.014	0.013	1.114	1	0.291	1.014	0.988	1.040
Male	0.193	0.197	0.965	1	0.326	1.213	0.825	1.785
Constant	−5.701	1.583	12.975	1	0.000	0.003		
Marijuana								
rsfMRI	−4.574	1.571	8.478	1	0.004	0.010	0.000	0.224
Age months	0.062	0.012	28.151	1	0.000	1.064	1.040	1.088
Male	0.367	0.166	4.879	1	0.027	1.444	1.042	2.001
Constant	−11.472	1.433	64.075	1	0.000	0.000		
Alcohol								
rsfMRI	1.036	0.478	4.703	1	0.030	2.818	1.105	7.188
Age months	0.021	0.003	44.747	1	0.000	1.022	1.015	1.028
Male	0.176	0.048	13.530	1	0.000	1.192	1.086	1.309
Constant	−3.338	0.385	75.257	1	0.000	0.036		

**rsfMRI =** ventral attention network (VAN) resting-state functional connectivity
